# Blocking N-Methyl-D-aspartic Acid (NMDA) Receptor Inhibits Heat-Sensitization Response of Moxibustion in Stroke Rats

**DOI:** 10.1155/2021/6463688

**Published:** 2021-08-26

**Authors:** Zhimai Lyu, Dandan Huang, Dingyi Xie, Yanjun Chen, Chunmei Wu, Rixin Chen, Weifeng Luo

**Affiliations:** ^1^Department of Neurology and Clinical Research Center of Neurological Disease, The Second Affiliated Hospital of Soochow University, Suzhou 215004, Jiangsu Province, China; ^2^Department of Neurology, The First Affiliated Hospital of Gannan Medical University, Ganzhou 341000, Jiangxi Province, China; ^3^Department of Basic Medical Sciences, Gannan Medical University, Ganzhou 341000, Jiangxi Province, China; ^4^Department of Acupuncture and Moxibustion, Affiliated Hospital of Jiangxi University of Chinese Medicine, Nanchang 330006, Jiangxi Province, China; ^5^Department of International Exchange and Cooperation, Jiangxi University of Chinese Medicine, Nanchang 330006, Jiangxi Province, China; ^6^Department of Health Statistics, School of Public Health & Health Management, Gannan Medical University, Ganzhou 341000, Jiangxi Province, China

## Abstract

Our previous studies demonstrated that effects of moxibustion heavily relied on heat-sensitization response, a specific sensation induced by moxibustion in the ill body. On the sensation, long-term potentiation (LTP) of prelimbic cortex was attributed to heat-sensitization responses. The N-methyl-D-aspartic acid (NMDA) receptor plays a key role in LTP induction; however, little is known about the role of NMDA receptor in heat-sensitization response. The present study investigated the role of NMDA receptor in heat-sensitization response, specifically, NMDA receptor was inhibited by competitive glutamatergic antagonist, (±)-3-(2-carboxypiperazin-4-yl)propyl-1-phosphonic acid (CPP), observing the frequency of heat-sensitization response in moxibustion treatment and evaluating the conducive outcomes to cerebral infarct rats for rehabilitation. Heat-sensitization response in cerebral infarct rats was regularly measured for all the samples when exposed to moxibustion. Intraperitoneal injection of CPP was conducted, and soon afterwards, a significant drop of heat-sensitization response in all the samples was measured. Moreover, moxibustion efficiency on rehabilitation was unfavourably affected in cerebral infarct rats when compared to vehicle injection control. This indicated that NMDA receptor antagonist made a negative impact on induction of heat-sensitization response and consequently affected cerebral infarct rats to rehabilitate under moxibustion treatment. It also suggested that activating NMDA receptor played a positive part in ischemic stroke rehabilitation, and regulating its activity could be a feasible way to increase heat-sensitization response, improving the effect of moxibustion.

## 1. Introduction

**Figure 1:**
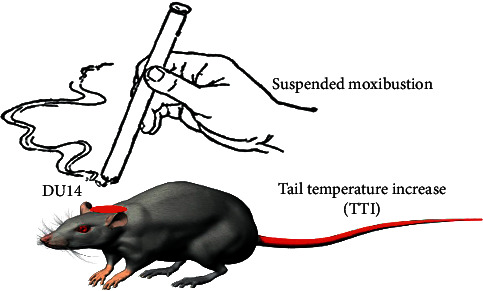
Heat-sensitization response in cerebral infarct rat. MCAO rats showed a tail temperature increase (TTI) when acupoint DU 14 was exposed to suspended moxibustion. This phenomenon reproduces the human beings' heat-sensitization response in cerebral infarct rats. The red region on the rat's neck represents the location of acupoint DU 14. The red shown on the rat's tail represents the temperature increasing.

Suspended moxibustion, a method of moxibustion treatments, is applied with heat generated from burning moxa 3–5 cm above over skin surface. During this process, healthy subjects usually perceive the local warmth upon moxibustion alone, while patients often complain of the heat-sensitization responses, including strong warmth spreading around the stimulating site or penetrating into the body, accompanied quite frequently with pleasant feelings, and better clinical treatment efficacy in a number of diseases [1, 2]. Further functional magnetic resonance imaging and electroencephalogram studies confirmed certain changes in certain cerebral regions accompanied with heat-sensitization responses, especially in prefrontal cortex. Noticeably, studies have demonstrated that long-term potentiation (LTP) of prelimbic cortex was attributed to heat-sensitization responses [3–5]. Since LTP induction depends critically on activation of N-methyl-D-aspartate (NMDA) receptors [6], we hypothesized that the NMDA receptor could be involved with heat-sensitization response. It may manifest in inhibiting the NMDA receptor when heat-sensitization responses occur. Should the response be eliminated by selectively blocking NMDA receptor in vivo, the hypothesis then will be asserted. Heat-sensitization responses were repeatedly observed in cerebral infarct rats when the acupoint dà zhuī (DU 14) being exposed to suspended moxibustion, with tail temperature increase (TTI) signifying the sensation, heat-sensitization response (Figure 1 exhibits the conduction and the responses) [7]. This study then investigated the frequency of TTI and moxibustion efficacy in cerebral infarct rats when NMDA receptor was inhibited.

## 2. Materials and Methods

### 2.1. Background

The compound that antagonizes the NMDA receptor in this study should be potent, highly selective, and administered systemically, so as to inhibit the NMDA receptor effectively in vivo. Among the compounds, (±)-3-(2-carboxypiperazin-4-yl)propyl-1-phosphonic acid (CPP), a competitive antagonist of NMDA receptor, has been described as a potent and highly selective NMDA antagonist in vivo [8] and is 5-fold more potent than AP5 or AP7. This compound antagonizes the NMDA receptor by reversibly binding to the glutamate binding site and shows no significant effect at kainate or quisqualate receptors [9]. Unlike most other competitive antagonists, CPP's penetration of the blood-brain barrier has led to its wide adoption and makes it effective when administered systemically in experiments [10]. CPP has already shown to block LTP induction effectively in vivo [11] and the dose of CPP (10 mg/kg) used in this study to the body temperature of rats can be ignored [12]. Therefore, CPP was used as an antagonist of NMDA receptor in this study.

### 2.2. Animals

Male Sprague-Dawley rats at the weight between 220 g and 250 g were used in the experiment. All the usages of animal in the experiment were conducted in accordance with NIH Guidelines and approved by the Animal Use and Care Committee for Jiangxi University of Traditional Chinese Medicine (No. JZLLSC2019-0156) for scientific purposes. Efforts were made to minimize the number and sufferings of the animals used.

### 2.3. Experimental Design

**Figure 2:**
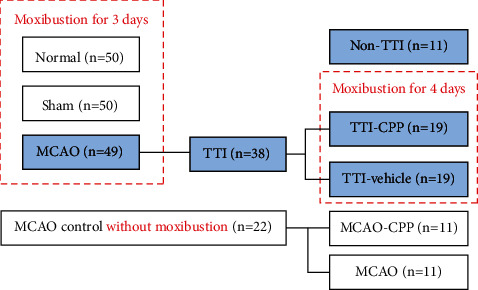
Flow chart depicting the experimental grouping. The number in the brackets is the final number of experimental rats.

150 rats were randomly stratified into 3 groups: (1) normal group (normal, *n* = 50), (2) sham-operation group (sham, *n* = 50), and (3) ischemia group (MCAO, *n* = 50). All the three groups were received moxibustion treatment for 3 days. According to the tail temperature change with moxibustion treatment, each group was further separated into two subgroups, including a nontail temperature increase subgroup (≤1°C an average of 3 days, non-TTI subgroup) and a tail temperature increase subgroup (>1°C an average of 3 days, TTI subgroup). Another group as control was comprised of 25 MCAO rats which were not put up with moxibustion (Figure 2).

### 2.4. Experimental Stroke in Rats

The middle cerebral artery occlusion (MCAO) rats were obtained as described previously [13]. Briefly, a fishing line (Simago Fishing Tackle Company) of 0.205 mm in diameter and 5 cm in length with a rounded tip was inserted through the common carotid artery and gently advanced to the origin of the middle cerebral artery in the anesthetized rats. After 2 hours of occlusion, the fishing line was withdrawn to allow for reperfusion. Sham-operated rats were treated in the same way, but the MCA was not occluded. The rats those died within 24 hours of the surgery having been done and the rats those performed nil in neurological deficit score all were excluded from selecting for further analysis. Core body temperature was monitored using a rectal probe throughout the operation and maintained at 37 ± 0.5°C by a heating lamp and a heating pad.

### 2.5. Suspended Moxibustion and Tail Temperature Measurement

The rats were individually placed in purpose-built cages which were designed for facilitating suspended moxibustion exercise and also for the rats situating in a comfortable position when the acupoint DU 14 was exposed to moxibustion treatment. Room temperature is 25 ± 2°C. The rat's midpoint tail temperature was recorded at interval time 2 minutes with electro-digital thermometer while being exposed to moxibustion treatment for 30 minutes.

### 2.6. Intraperitoneal Injection of NMDA Receptor Antagonist CPP

MCAO rats in TTI subgroup further received different treatments: TTI-vehicle received intraperitoneal injection of 0.9% saline and TTI-CPP received 10 mg/kg CPP in saline. The dose of CPP (10 mg/kg) had no sedative effects in rats and did not affect the body temperature of rats [12]. One hour after that, 30 min moxibustion on acupoint DU 14 was carried out once a day for another 4 days and the tail temperature was also measured. Simultaneously, about half of MCAO rats in the control group were received intraperitoneal injection of CPP as parallel control (MCAO-CPP) (Figure 2).

### 2.7. Neurological Assessment

Neurological functions were assessed at 0 h, 1 d, 3 d, and 7 d after MCAO. The assessments were based on a previous description [14] and conducted without bias, i.e., the rats were only labeled with codes and without their group identification.

### 2.8. Statistical Analysis

Chi-square tests were used to compare ratios between groups. Quantitative variables were analyzed with one-way analysis of variance with *post hoc* Newman–Keuls multiple range test for multiple groups. SPSS 20.0 was used for analysis. *P* values under 0.05 were considered statistically significant.

## 3. Results and Discussion

### 3.1. Analysis of Tail Temperature and Subgroup Classification

**Figure 3:**
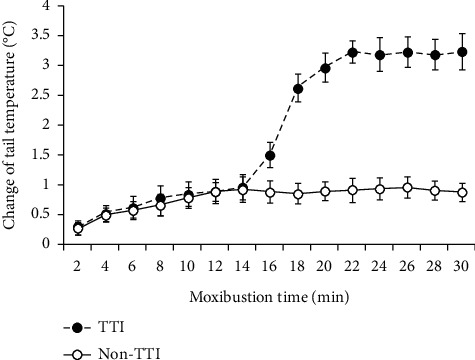
Tail temperature change following suspended moxibustion. Tail temperature began to quickly increase immediately after suspended moxibustion. At about 5–10 min, the change of tail temperature in non-TTI subgroup reached a relatively stable level but less than 1°C on average. However, the tail temperature of TTI subgroup continued to increase to a peak value, which was maintained until the end of the treatment at 30 min. The change of tail temperature in TTI subgroup is about 3°C. Data coming from the tail temperature change of all 149 rats on the second moxibustion day are presented as a representative. Data are expressed as mean ± SD.

Having excluded the ‘incompetent' rats, eventually, 50 normal, 50 sham, and 49 MCAO rats were treated with 30 min moxibustion once a day for 3 days. 10 out of the 50 normal rats (20%), 12 out of the 50 sham rats (24%), and 38 out of the 49 MCAO rats (77.55%) were observed to show a stable TTI on the third moxibustion day (Table 1). The results were consistent with the clinical observations in human patients, i.e., the frequency of heat-sensitization response was significantly increased in the ill body than those who were not in any medical conditions. As to some MCAO rats without TTI under moxibustion treatment, the sensitive location might not be the acupoint DU 14 in those MCAO rats, though the acupoint DU 14 was the high-frequency location that could be stimulated to induce TTI in MCAO model. Noticeably, a small part of rats in normal or sham rats exhibited TTI. Similar circumstances were observed in our clinical practice, e.g., the frequency of heat-sensitization responses was about 10–20% in healthy people [1], which is much lower than that of subjects under medical conditions. It might be related to congenital factors, or perhaps those so called healthy subjects are not as healthy as we considered. There were a lot of people in the above 10–20% healthy people classified into subhealth category when diagnosing by Chinese medicine such as pulse diagnosis or tongue diagnosis. In addition, we also observed that a small number of people considered to be in healthy condition showed powerful heat-sensitization responses with familial clustering during moxibustion. We consider that this phenomenon provoked further study on the relationship between genetic inheritance and heat-sensitization responses. The change of tail temperature in TTI subgroup was about 3°C while it was less than 1°C on average in non-TTI subgroup (Figure 3). Accordingly, 38 MCAO rats with TTI were randomly and equally selected for further intraperitoneal injection subgroups: TTI-vehicle and TTI-CPP. 22 “competent” MCAO rats without moxibustion were used as control; of these, 11 MCAO rats were randomly selected to receive intraperitoneal injection of CPP (MCAO-CPP).

### 3.2. The Frequency of TTI following Intraperitoneal Injection of CPP

19 TTI-vehicle rats showed TTI and the frequency remained the same over the following four consecutive days. While 19 TTI-CPP rats did not show TTI on the first and second days, but resumed TTI from the third day in sequence that was dependent on individual metabolism (Table 2). The effect of TTI eliminated in TTI-CPP rats was considered the results of blocking NMDA receptors by CPP and thereby preventing LTP. This indicated that NMDA receptor was involved in heat-sensitization response and the antagonist had negative impact on induction of heat-sensitization response. As the heat-sensitization response (or TTI) was closely related to the subhealthy people or patients and varied with the state of a disease or the kind of disease [1], additional experimental injuries may affect the induction of TTI in MCAO rat. Therefore, it is necessary to reduce additional experimental traumas in MCAO rats as much as possible. Since CPP can pass through the blood-brain barrier [15], intraperitoneal injection was preferred to use in this study, instead of lateral ventricle injection or intrathecal injection that need higher experimental operation requirements and will result in additional injuries, specifically, lateral ventricle injection requests drilling a cranial hole and microsyringe needling. The cortex will be pierced under these operations.

### 3.3. Neurological Deficit Scores

**Figure 4:**
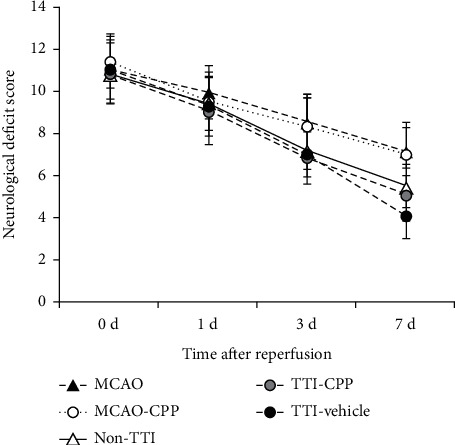
Neurological assessment in MCAO rats with or without moxibustion treatment. Neurological deficit scores were graded on a scale of 0 to 18 (normal score, 0; maximal deficit score, 18). Data are presented as mean ± SD. There were no differences between all subgroups on day 0 and day 1. Day 3: non-TTI *vs*. MCAO or MCAO-CPP, *P* < 0.05; TTI-CPP *vs*. MCAO or MCAO-CPP, *P* < 0.001; TTI-vehicle *vs*. MCAO or MCAO-CPP, *P* < 0.01. Day 7: non-TTI *vs*. MCAO or MCAO-CPP, *P* < 0.01; TTI-CPP *vs*. MCAO or MCAO-CPP, *P* < 0.001; TTI-vehicle *vs*. MCAO or MCAO-CPP, *P* < 0.001; TTI-vehicle *vs*. non-TTI, *P* < 0.01; TTI-vehicle *vs*. TTI-CPP, *P* < 0.05.

As to neurological assessment in the following subgroups (1) MCAO (*n* = 11), (2) MCAO-CPP (*n* = 11), (3) non-TTI (*n* = 11), (4) TTI-CPP (*n* = 19), and (5) TTI-vehicle (*n* = 19), the results showed that (1) non-TTI, TTI-CPP, and TTI-vehicle subgroups showed having ameliorated neurological deficits significantly compared to MCAO and MCAO-CPP subgroups from the 3 d; (2) there were no difference between non-TTI and TTI-CPP subgroups, and no difference between MCAO and MCAO-CPP subgroups as well; (3) TTI-vehicle subgroup reduced neurological deficit score markedly compared to the non-TTI and TTI-CPP subgroups on the 7 d (Figure 4). According to the above results, single intraperitoneal injection of CPP presumably would not affect the rehabilitation of MCAO rats without moxibustion when compared to MCAO controls. These outcomes clearly demonstrated that NMDA receptor antagonist CPP affected moxibustion efficiency for rehabilitation of MCAO rats. Although blocking NMDA receptors helped alleviating the acute injury of cerebral infarction through reducing the rise of intracellular calcium [16], the same operation indeed weakened the neuroplasticity by inhibiting LTP in the stage of rehabilitation for cerebral infarction [17]. The function of NMDA receptor in different stages of cerebral infarction is different [18]. It controls critical events in the formation and development of synaptic plasticity during stroke rehabilitation. Activating NMDA receptor played a prosurvival role, which was also presented in others' postacute phase stroke studies [17, 19], in stroke rehabilitation under moxibustion. In addition, as the above results illustrated that the frequency of heat-sensitization response (or TTI) was related to the rehabilitation, further biochemical methods such as 2,3,5-triphenyltetrazolium chloride histology to test the efficacy are not necessary, so as to minimize the sufferings of the animals as much as possible.

## 4. Conclusions

The links between NMDA receptor and LTP on the one hand and LTP with heat-sensitization response on the other suggested that NMDA receptor was involved in heat-sensitization response. The effects observed in this study proved the association. It found the antagonist CPP inhibited NMDA receptor to perform in responses to moxibustion, which resulted in attenuating the moxibustion effect, with decreased heat-sensitization response. This suggested that activating NMDA receptor played a pivotal role in the induction of heat-sensitization response. The results also suggested that adjusting heat-sensitization response via modulating the activity of NMDA receptors by pharmacological tools might be a feasible way to improve the efficacy of moxibustion.

## Figures and Tables

**Table 1:** The frequency of TTI in moxibustion treatment groups.

Day	Group	TTI (*n*)	Non-TTI (*n*)	Total (*n*)	TTI/total (%)
1	Normal	10	40	50	20.00
	Sham	11	39	50	22.00
	MCAO	27	22	49	55.26

2	Normal	10	40	50	20.00
	Sham	12	38	50	24.00
	MCAO	38	11	49	77.55

3	Normal	10	40	50	20.00
	Sham	12	38	50	24.00
	MCAO	38	11	49	77.55

**Table 2:** The frequency of TTI following intraperitoneal injection of CPP in MCAO rats with moxibustion treatment.

Day	Group	TTI (*n*)	Non-TTI (*n*)	Total (*n*)	TTI/total (%)
1	Vehicle	19	0	19	100.00
	CPP	0	19	19	0

2	Vehicle	19	0	19	100.00
	CPP	0	19	19	0

3	Vehicle	19	0	19	100.00
	CPP	2	17	19	10.53

4	Vehicle	19	0	19	100.00
	CPP	6	13	19	31.58

## Data Availability

The data used to support the findings of this study are available from the corresponding author upon request.

## Abbreviations

NMDA: N-Methyl-D-aspartic acid
LTP: Long-term potentiation
TTI: Tail temperature increase
MCAO: Middle cerebral artery occlusion
CPP: (±)-3-(2-Carboxypiperazin-4-yl)propyl-1-phosphonic acid
DU 14: Acupoint dà zhuī.
